# The cannulation strategy in surgery for acute type A dissection

**DOI:** 10.1007/s11748-016-0711-7

**Published:** 2016-09-20

**Authors:** Tomonobu Abe, Akihiko Usui

**Affiliations:** Department of Cardiac Surgery, Nagoya University Graduate School of Medicine, 65 Tsurumai-cho, Showa-ku, Nagoya, Aichi 466-8550 Japan

**Keywords:** Aorta, Dissecting aneurysm, Cardiopulmonary bypass, Axillary artery, Femoral artery

## Abstract

The rates of mortality and morbidity remain high in surgery for acute type A dissection. There is controversy regarding the best cannulation strategy for achieving good clinical results. Each cannulation technique has different anatomical characteristics and a different flow pattern inside the aorta during cardiopulmonary bypass. Some adverse, clinically important outcomes may be related to events at this time. Femoral artery cannulation, axillary artery cannulation, and central aortic cannulation are the three major cannulation strategies that are adopted in many centers in the world. Accumulating results from comparative studies between right axillary artery cannulation and femoral artery cannulation show that right axillary artery cannulation is associated with better clinical outcomes. However, all of the studies have been retrospective, and few studies have compared the results of other combinations of cannulation strategies. Observational studies using newer monitoring techniques clearly show that no perfusion strategy is perfect or free from complications. In summary, the evidence is insufficient to make a strong recommendation regarding cannulation strategies. Based on the fairly consistent results of retrospective studies, more surgeons are tending to switch from a retrograde perfusion strategy to adopt an antegrade perfusion strategy. Regardless of the routine cannulation strategy that is adopted, careful monitoring and a swift response to adverse events are necessary. The further accumulation of evidence is warranted.

## Introduction

The aim of this review is to provide an overview of the cannulation strategies that have been adopted for acute type A dissection repair.

**Fig. 1:**
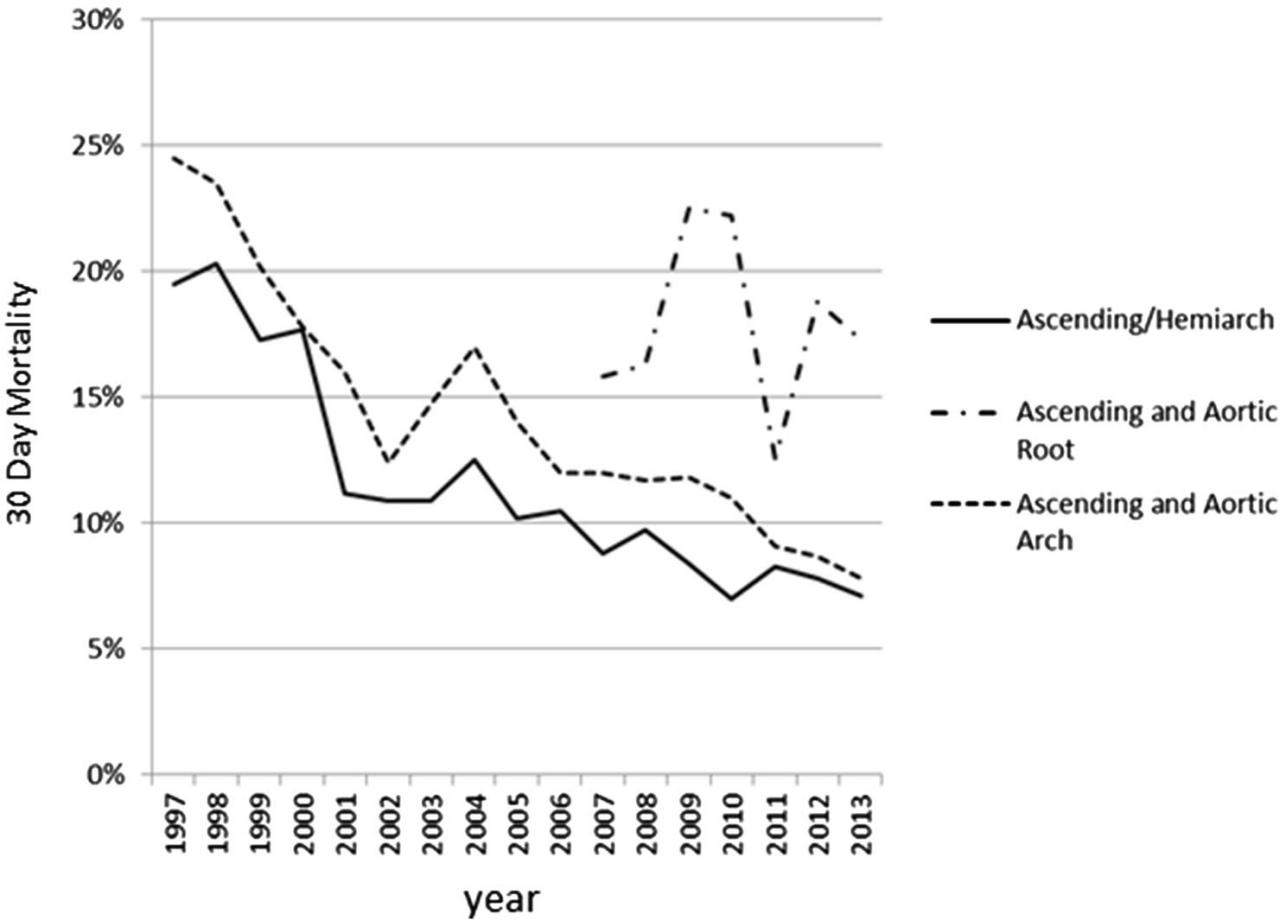
30-day mortality rate of surgery for acute type A dissection in the Japanese Association for Thoracic Surgery annual surveys. Ascending/Hemiarch, replacement of the ascending aorta and hemiarch replacement; ascending and aortic root, replacement of the ascending aorta and aortic root; ascending and aortic arch, replacement of the ascending aorta and aortic arch

Surgery for acute type A dissection is a complex topic [1, 2]. It is not complex in terms of the surgical maneuvers involving the aorta itself—standard hemiarch replacement only consists of two anastomoses. Surgery in acute type A dissection is complex mostly due to the adjunctive methods. Although some data have shown improving (Fig. 1) [3], the rate of operative mortality remains high. The in-hospital mortality rate reported in the international registry of acute aortic dissection (IRAD) [4] was 18–25 %, and that in the Japanese Association for Thoracic Surgery annual surveys in 2013 was 9.1 % [3]. Many efforts have been made to improve outcomes. There are many elements in surgery, such as temperature, open anastomosis, cerebral protection, visceral organ protection, and the cannulation strategy.

Although this review primarily concentrates on cannulation strategies, all of the elements in surgery are interrelated. To avoid losing sight of the big picture, we will first briefly describe the principles and the overall surgical techniques of contemporary surgery for acute type A dissection. We will then list the cannulation strategies and describe their characteristics and review the results of comparative studies of cannulation strategies. Finally, we will describe various special situations.

## The principles and techniques of surgery for acute type A dissection

### The principles of surgery for acute type A dissection

Acute type A aortic dissection is associated with a high rate of short-term mortality. Sixty percent of patients die within 30 days if they do not undergo surgery [1, 5]. In 40–70 % of fatal cases, cardiac tamponade or rupture is the cause of death [6, 7]. Visceral ischemia accounts for 13.9 % of fatal cases and is the second most common cause of death [6, 7]. The prior aim of surgery in the acute phase is to save patients’ lives [2, 5, 8]. In the majority of patients with type A aortic dissection, the intimal tear is located in the ascending aorta. The standard surgery is the replacement of the ascending aorta and proximal arch to reduce the risk of cardiac tamponade or rupture by preventing the proximal extension of dissection [1, 2, 5]. This also serves to redirect the aortic blood flow to the true aortic lumen, and is therefore, likely increase the blood flow in the aortic branches that were previously compromised.

To achieve a successful repair, the extremely fragile dissected aortic layers must be reconstructed proximally and distally. The current consensus favors open distal anastomosis, which allows for meticulous distal aortic anastomosis and the prevention of injuries by usual aortic clamping [5, 9]. Hypothermia and circulatory arrest are necessary for this strategy.

When aortic valve regurgitation is present, aortic valve competence must be restored. If the aortic arch contains an intimal tear, resection of the intimal tear by arch replacement is necessary.

### The surgical techniques for acute type A dissection

The usual preparations are made for operations involving cardiopulmonary bypass (CPB). Median sternotomy is performed.

Initial arterial cannulation is performed. This is the main subject of the present article, and the surgical techniques of each cannulation strategy will be discussed later in the “Cannulation strategies”. The right atrium or the venae cavae are cannulated, and CPB is established. The patient is cooled for hypothermic circulatory arrest. The lowest core temperature differs among institutions from 28 °C to less than 20 °C [10, 11]. Some surgeons clamp the aorta before reaching the target temperature then make preparations and perform proximal anastomosis. Some surgeons are strongly opposed to this [2, 12], and do not touch the ascending aorta until the target temperature is reached.

When the patient’s body temperature is sufficiently low, the pump is stopped, and the ascending aorta is opened. The patient is under circulatory arrest. The brain protection strategy during this period differs widely among institutions. There are three possible strategies: simple hypothermic circulatory arrest, retrograde cerebral perfusion, and antegrade selective cerebral perfusion [2, 13]. Antegrade cerebral perfusion can be either unilateral or bilateral. Unilateral selective cerebral perfusion is done by clamping the origin of the right innominate artery and perfusing via the right axillary artery. Bilateral selective cerebral perfusion is performed using balloon tip catheters [14]. Regarding myocardial protection, some institutions use antegrade selective cardioplegia, while others use retrograde cardioplegia or both [5, 15].

Under circulatory arrest, the distal aorta is trimmed and distal anastomosis is performed. An aortic wall affected by dissection is vulnerable, necessitating a particularly meticulous anastomosis. Various anastomotic options to reinforce friable aortic tissue such as sandwich technique with Teflon felt strips, adventitial inversion technique, and use of glues, have been described [16]. All of these techniques are somewhat time-consuming, and anastomosis generally takes longer in acute aortic dissection than in chronic aneurysms. Cardiopulmonary bypass is re-established via the graft anastomosed to the distal end [5]. This re-initiation of CPB with antegrade arterial perfusion was explained to be important because retrograde perfusion at this stage might result in pressurizing the false lumen [17]. The rewarming of the patient is then initiated. Proximal anastomosis is performed. The aortic graft is unclamped. After the completion of rewarming, the pump is weaned off. Hemostasis is achieved, the cannulas are removed and the chest is closed.

## The general considerations about cannulation strategies

In this study, the term “cannulation strategy” refers to initial cannulation for the establishment of CPB and for cooling until hypothermic circulatory arrest. It has generally been advised that arterial blood flow should be re-started from the graft as antegrade aortic flow after the completion of distal anastomosis [5, 9, 17]. The surgery after distal anastomosis is thus essentially the same, irrespective of the cannulation strategy that is adopted.

It may be useful to consider why the cannulation site for this short period of time, less than 1 h, would possibly make difference in the clinical outcomes. Theoretically, four aspects differ among the cannulation strategies. These include malperfusion during the cooling period, the time needed to establish cardiopulmonary bypass, possible differences in the brain protection strategy during circulatory arrest, and the possible exacerbation of aortic dissection during cannulation and the cooling period.

### Malperfusion during the cooling period

End-organ malperfusion is reported to occur in 16–33 % of patients with acute type A aortic dissection. There are two types of intraoperative malperfusion during CPB. One is the persistence of preoperative malperfusion; the other is new malperfusion that occurs after the initiation of CPB. Malperfusion may involve any of the major aortic branches and has the potential to result in coronary, brain, spinal cord, visceral organ, or limb ischemia [18–20]. Stroke and malperfusion are important causes of operative death. [21, 22]. Although ischemic stroke can occur at any stage of surgery and may be attributed to other reasons than malperfusion, such as preoperative shock or prolonged circulatory arrest, many surgeons believe that some strokes occur by malperfusion during the period in which the cannulation strategy is most important.

Many surgeons who favor axillary cannulation and central cannulation believe that the antegrade perfusion strategy is advantageous in preventing brain malperfusion [1, 23] although there are opposing views [23]. Pre-existing stroke is a difficult situation, and will be discussed in “Special considerations in cannulation strategies” section.

### The time needed to establish CPB

Preoperative shock has always been the strong risk factor of operative death [24, 25]. The quicker establishment of cardiopulmonary bypass may result in improved outcomes in these patients. Generally speaking, axillary cannulation is more time-consuming than femoral artery cannulation [23, 26]. Central aortic cannulation by the Seldinger technique and transapical cannulation can be completed quickly [27, 28].

### The exacerbation of aortic dissection during the cooling period

The freshly dissected aortic intima and adventitia are extremely fragile. The dissection of the aortic wall is prone to extend distally and proximally. The potential risk of exacerbating the dissection and the potential risk of rupture are concerns that are expressed regarding central aortic cannulation [29, 30].

Some surgeons believe that pressurizing the false lumen by aortic clamping and retrograde perfusion may result in the development of a new intimal tear [17].

### Possible differences in brain protection strategies

Brain protection during circulatory arrest is another important issue in the surgical techniques for the treatment of acute type A aortic dissection. Many surgeons who routinely use right axillary artery cannulation use the arterial cannula as an arterial route to the brain in the antegrade cerebral perfusion strategy [14]. Some studies have actually shown that antegrade cerebral perfusion was more frequently used with axillary artery cannulation [31]. This difference makes it difficult to distinguish the effects of the cannulation strategy from the brain protection strategy. In addition, right axillary artery cannulation is often specifically related to unilateral antegrade perfusion [32].

## Cannulation strategies

Femoral artery cannulation, axillary artery cannulation, and central aortic cannulation are probably the most widely adopted strategies in the world [33]. Transapical cannulation will also be discussed. The theoretical advantages and disadvantages of each of these strategies are summarized in Table 1.

**Table 1:** The advantages and disadvantages of each cannulation strategy

	Advantage	Disadvantage
Femoral artery cannulation	Quick to establish CPB Easy to access even with closed chest Less likely to be dissected	Possible more malperfusion due to retrograde aortic flow Possible atherosclerotic emboli
The right axillary artery cannulation	Antegrade flow Can be used for antegrade cerebral perfusion rout	More time-consuming Technically demanding in some cases Possible injury to the brachial nerves
Central aortic cannulation	Antegrade flow Quick to establish CPB	Possible false lumen perfusion Possible aortic rupture
Transapical cannulation	Antegrade flow Quick to establish CPB Less likely to cause aortic rupture	Technically unfamiliar to many surgeons Dangerous in patients with aortic stenosis

*CPB* cardiopulmonary bypass

### Femoral artery cannulation

The femoral artery has been the cannulation site of choice for a number of years [5], and still is in many institutions, including centers that are renowned for aortic surgery [34]. Some centers have described their extensive experience with femoral cannulation and shown good results [23].

The common femoral artery is typically located slightly medial and inferior to the midpoint of the inguinal ligament. Wide exposure of the femoral vessels is best obtained through a vertical incision [35]. When limited exposure is thought to be enough for arterial cannulation, oblique incision may be selected [35]. When cannulating, the open Seldinger technique may be beneficial because it is quick, requires minimal dissection and manipulation of the femoral artery, and allows maintenance of limb perfusion [34]. Exposing the femoral arteries is relatively easy. Cardiopulmonary bypass can be established quickly; thus femoral cannulation is considered to be advantageous in hemodynamically unstable patients [26, 36]. The theoretical disadvantage of femoral artery cannulation is that stroke and malperfusion, which may be caused by dynamic obstruction, may occur more frequently with femoral cannulation than with other strategies that provide antegrade aortic flow. Embolic complications caused by atheromatous emboli are also possible. Several comparative studies have shown that femoral artery cannulation is associated with higher mortality and stroke rates than axillary artery cannulation [33].

Etz et al. showed that femoral cannulation was only associated with worse results in patients with distal entry, and that it was associated with good results as “antegrade perfusion” in patients with proximal entry [37].

Severe atherosclerosis in the thoracoabdominal aorta, iliofemoral system, or distal arch on preoperative computed tomography can be a reason not to choose femoral cannulation [34]. The femoral artery with a dissection flap extending to it is not usually chosen as a cannulation site [34].

### Axillary artery cannulation

Axillary artery cannulation was introduced in the late 1990s [14, 38–41]. Recently, this technique seems to be gaining wider acceptance [33].

There are several anatomical approaches to the axillary artery that is used for cannulation in aortic surgery. The infraclavicular approach is the most proximal approach for exposing the first part of the axillary artery [35, 42]. Under this approach, an 8 cm horizontal skin incision is made below the clavicle, and the pectoralis major muscle is split. The neurovascular bundle is located in the adipose tissue deep in the clavipectoral fascia. The deltopectoral approach is also used to expose the second and third parts of the axillary artery for cannulation [41]. Small incisions in the axilla are also reported [14, 39]. The axillary artery can be directly cannulated or perfused via a piece of vascular graft, which is anastomosed in an end-to-side fashion.

The theoretical advantages of this technique are as follows: first, it can provide antegrade aortic flow during the cooling period; and second, it can be used as an infusion route to the brain in an antegrade cerebral perfusion strategy during circulatory arrest, simply by blocking the innominate artery. The disadvantage of this technique is that the exposure is more time-consuming, especially in obese patients. It can be a clinically important drawback in patients who are hemodynamically unstable. The axillary artery can be exposed within a few minutes by approaching the axilla [14]; however, only smaller caliber cannulas can be inserted. Another disadvantage is the possibility of injury to the brachial nerves [14].

### Central aortic cannulation

Central cannulation to the true lumen of the ascending aorta in acute type A aortic dissection was introduced in the early 2000s [28, 43].

The ascending aorta is exposed via median sternotomy. An advantage of this cannulation strategy may be the fact that an extra incision is unnecessary. Although experienced surgeons rarely have this problem, both subclavian and inguinal incisions are associated with some risk of wound complications. Locating an adequate site for cannulation is of paramount of importance since the sequence of false lumen cannulation can be catastrophic. Computed tomography, transesophageal echo, and direct epiaortic echo are to obtain precise anatomical information [44, 45]. It can either be cannulated using the Seldinger technique or directly by purse-string stitches [28, 43–45]. Frederick et al. claimed that true lumen cannulation is possible even in patients with circumferential dissection under real-time transesophageal echo guidance [44]. Good results have been reported with central aortic cannulation [44–46]. The theoretical advantages of this strategy are that antegrade perfusion is achieved and that only a short time is necessary to establish CPB. The major concerns in relation to the use of this technique are the rupture of the cannulation site and false lumen perfusion [29, 30, 47].

### Transapical cannulation

Transapical cannulation for acute type A aortic dissection was introduced in the 2000s and was followed by studies of a large series of patients [48, 49]. Caval cannulation is done, first [49]. A 1-cm incision is then made at the apex of the left ventricle without a purse-string suture, and a cannula with curved stylet is inserted through the apex and across the aortic valve until positioned in the ascending aorta under transesophageal echocardiographic guidance [49]. It took only short period of time for CPB to be established [27]. Good results have been reported with this technique [50, 51]. The following advantages of this technique were described: antegrade perfusion, true lumen perfusion, and that cannulation can be performed in a short period of time. The lower possibility of aortic rupture in comparison to direct aortic cannulation may also be included as an advantage [49]. The technique should not be used for patients who have aortic stenosis [49]. Bleeding at the cannulation site is not usually a problem [49].

## Comparative studies on cannulation strategies in surgery for acute type A dissection

**Fig. 2:**
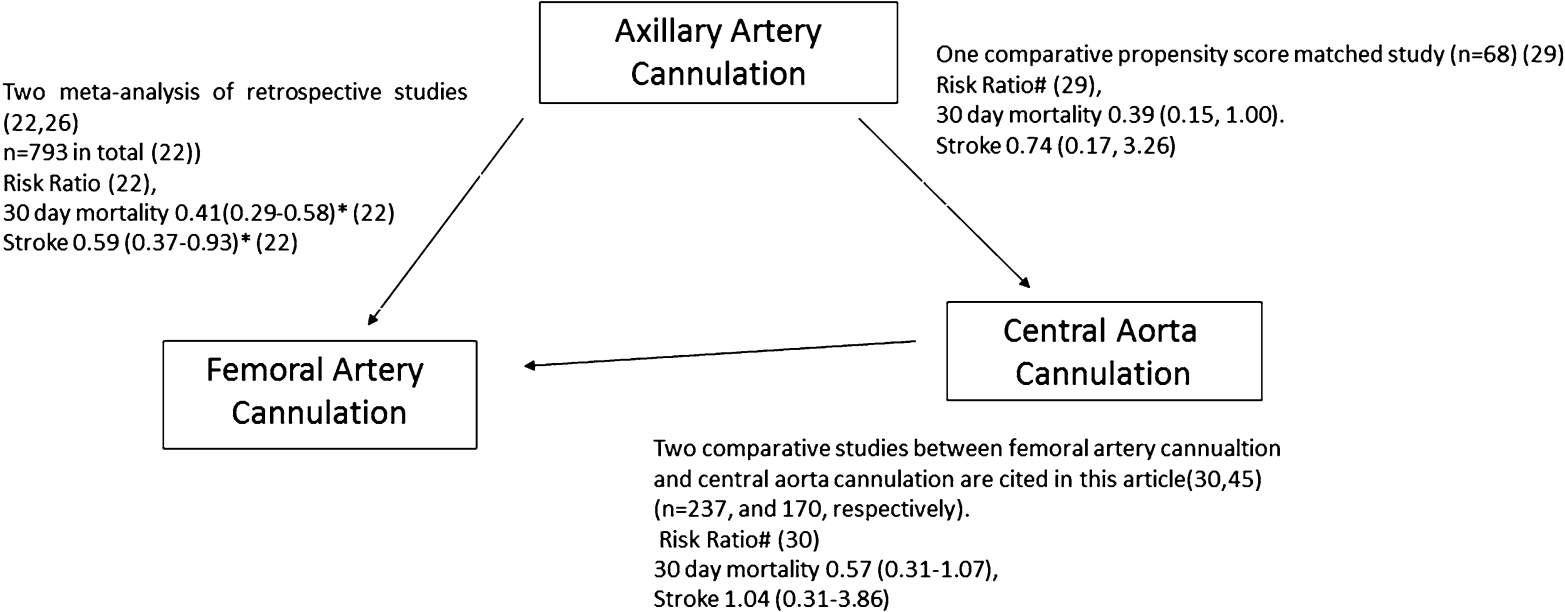
Schematic illustration of comparative studies in cannulation strategy in surgery for acute type A dissection. * *p* < 0.05; ^#^Risk ratio was calculated by authors from published data. Note, this is not a formal report of network meta-analysis. Literature search was not systematically done

The clinical outcomes of the different cannulation strategies have been reported comparative analyses. There have been no prospective randomized controlled studies on this subject. This situation is completely understandable when one considers that acute type A dissection requires emergent treatment, and that its treatment requires a high level of expertise. Retrospective studies covering the major clinical outcomes and their meta-analyses are therefore considered to provide the highest level of evidence regarding this topic at this time. The results should be interpreted with caution due to the retrospective nature of the included studies. A casual schematic illustration of the comparative studies cited in this article is shown in Fig. 2.

### Femoral artery cannulation vs. axillar artery cannulation

There have been two meta-analyses of retrospective observational studies [26, 31]. Ren et al. extracted data from 9 studies, while Benedetto extracted data from 8 studies. Six of the studies were included in both papers.

Regarding the rates of in-hospital mortality and neurological deficit, both papers reached the same results. The rate of in-hospital mortality was lower in patients who were cannulated via the right axillary artery. The risk ratio was 0.41 with a 95 % confidence interval of 0.29–0.58 [31]. The rate of permanent neurological deficit was also lower in the patients who received axillary artery cannulation. The risk ratio was 0.59 (95 % confidence interval [CI], 0.37–0.93). Interestingly, Ren et al. showed no difference in the rate of malperfusion (brain, limb, and visceral) (Odds Ratio 0.64; 95 % CI 0.37–1.90).

The authors of the both studies were aware of two possible sources of bias. One is the preoperative condition of the patients; the other is the difference in the brain protection strategies.

Femoral artery cannulation is more often used in hemodynamically unstable patients. Benedetto et al. performed a multivariable meta-analysis that included variables such as preoperative shock, and showed that axillary artery cannulation was an independent protective factor of both in in-hospital mortality and permanent neurological deficit.

Another important possible bias is the different brain protection strategies. Benedetto et al. performed a sub-group analysis of studies in which the same single brain protection strategies were employed, and found similarly better results in the axillary artery cannulation group.

Based on the fairly consistent results of retrospective studies, more surgeons seem to be switching from a retrograde perfusion strategy to adopt an antegrade perfusion strategy [33].

Etz et al. reported that the difference between the antegrade perfusion strategy and the retrograde perfusion strategy was only observed in patients with distal entry, suggesting that a patient-specific approach might be possible [37].

### Central aortic cannulation vs. femoral artery cannulation

Several studies have compared central aortic cannulation vs. peripheral cannulation [28, 30, 47, 52]. Reese et al. showed that the rates of operative mortality and myocardial infarction were lower in patients who had undergone central cannulation than they were in patients who underwent peripheral cannulation in a population of 70 patients [28]. Other studies showed negative results. In a population of 170 patients, Klotz et al. did not find significant differences in any of the clinically important outcomes between central cannulation and femoral cannulation [47]. Kamiya et al. showed no differences in a population of 237 patients [30]. Three of five cited studies included axillary artery cannulation in peripheral artery cannulation group [28, 52]. The studies of Kamiya et al. and Klots et al. are the only two studies that compared exclusively central aortic cannulation and femoral artery cannulation [30, 47].

### Central aortic cannulation vs. axillary artery cannulation

Sabashnikov et al. compared their results of axillary artery cannulation and central cannulation. This study seems to be the only comparative study with a significant number of patients that compares these two strategies [29]. They compared the results in a total of propensity-matched patients. The axillary artery cannulation group showed a trend towards lower 30-day mortality (RR 0.37; 95 % CI 0.15–1.00, calculated by authors from published data). There was no difference in the incidence of stroke (RR 0.74; 95 % CI 0.17–3.26, calculated by the authors from published data). Long-term survival was significantly worse in patients with central aortic cannulation than in those with axillary artery cannulation [29].

### Transapical cannulation vs. peripheral cannulation

Two studies compared the short-term results of transapical cannulation and peripheral (femoral and axillar artery) cannulation. Djukanovic showed no difference in the rates of mortality, stroke, myocardial infarction, or renal insufficiency in 111 patients. Suenaga reported no difference in rates of mortality or stroke in 80 patients. They showed that the time from skin incision to CPB was shorter with transapical cannulation than it was with femoral cannulation [50].

## Special considerations in cannulation strategies

### The alteration and addition of arterial cannulation in intraoperative malperfusion

Two types of intraoperative malperfusion occur during CPB. Preoperative malperfusion may persist after CPB is commenced. New malperfusion may also occur after the initiation of CPB [53].

Preoperative malperfusion is a difficult situation. When patients are brought to an operating room for emergency central repair, the restoration of malperfusion is expected in majority (90 %) of patients after they have been placed on CPB, because true lumen flow usually increases [53–55]. In some cases, however, malperfusion persists [53, 54].

New malperfusion can also occur after the initiation of CPB, probably due to a dynamic obstruction mechanism [53, 56]. Orihashi et al. reported that intraoperative malperfusion occurs less often with axillary artery cannulation than with femoral artery cannulation [57]. However, even with a routine axillary artery cannulation strategy, intraoperative malperfusion occurred in 8.5 % of patients [53].

New intraoperative monitoring techniques such as transesophageal echo and the measurement of the brain oxygen saturation by near-infrared spectroscopy are being adopted in more institutions [57]. Surgeons and anesthesiologists have more chances to catch ominous signs. Since malperfusion syndrome is strongly associated with poor post-operative outcomes, every effort should be made to solve the problem. Although there is no simple formula for dealing with intraoperative malperfusion, the alteration or addition of arterial cannulation to different sites has been attempted, especially when a dynamic mechanism is suspected [53, 54, 57]. One of the uncannulated access sites among both axillary arteries, both femoral arteries, and the central proximal aorta will be cannulated as an alternative cannulation site based on the surgeon’s speculation about the mechanism of malperfusion. However, response is often unpredictable, and trial and error may be required.

Orihashi et al. described three possible treatments for malperfusion: the modification of the perfusion route, selective perfusion or the aspiration of the thrombus (or both), and revascularization [53].

### Carotid artery cannulation in cerebral malperfusion

Malperfusion involving the carotid artery is a serious situation, which often presents preoperatively as ischemic stroke or coma. Although the acceptable results of immediate central aortic repair have been reported with routine central aortic repair without any special methods [58–60], a significant number of patients remain disabled, and some have shown an exacerbation and eventual death [59, 61]. Some institutions are attempting carotid artery cannulation either by direct cannulation or by end-to-side graft anastomosis to the carotid artery to quickly and safely restore the blood flow [62–65]. The graft sewn to the carotid artery can be used for aorto-carotid extraanatomical bypass in cases of static obstruction [63, 65]. Good results have been shown. Since there were no controls in any of these studies, the clinical benefits of this strategy remain uncertain.

## Conclusion

Femoral artery cannulation, axillary artery cannulation, and central aortic cannulation are likely the three major cannulation strategies in surgery for acute type A dissection. Each of the different cannulation strategies has its advantages and disadvantages. Two meta-analysis comparing axillary artery cannulation and femoral artery cannulation show that axillary artery cannulation is associated with better results. However, all of the included studies were retrospective, and there was little evidence about other strategies. We conclude that the evidence is insufficient for making any strong recommendations regarding the cannulation strategy, although more surgeons seem to be switching from a retrograde perfusion strategy to an antegrade perfusion strategy based on the fairly consistent results of retrospective studies as well as theoretical advantages.

No cannulation strategy can be completely free from the risk of intraoperative malperfusion. The multimodal real-time monitoring of organ malperfusion has an important role. Certain precise analyses are identifying specific patient groups who show a particular benefit from certain cannulation strategies. These data may make a patient-specific approach possible in the future. The accumulation of further evidence is warranted.
